# ﻿New earthworms of the genus *Pheretima* (Clitellata, Megascolecidae) from Mount Bulusan, Sorsogon Province, Philippines

**DOI:** 10.3897/zookeys.1253.158651

**Published:** 2025-09-24

**Authors:** Yong Hong, Samuel W. James

**Affiliations:** 1 Department of Plant Medicine, College of Agriculture and Life Sciences, Jeonbuk National University, Jeonju 54896, Republic of Korea Jeonbuk National University Jeonju Republic of Korea; 2 Department of Regenerative Organic Agriculture, Maharishi International University Fairfield, Fairfield, Iowa 52557, USA Maharishi International University Fairfield Fairfield United States of America

**Keywords:** Biodiversity survey, *
sangirensis*-group, soil fauna, Southeast Asia, taxonomy

## Abstract

Three new species of the genus *Pheretima* are described from Mount Bulusan, Sorsogon Province, Luzon Island, Philippines: *Pheretima bulusanensis***sp. nov.**, *Pheretima sorsogonensis***sp. nov.**, and *Pheretima blackbirdensis***sp. nov.** All are in the *sangirensis*-group of Sims and Easton (1972), which is characterized by having one pair of spermathecal pores in 7/8. The three new species have equatorial unpigmented stripes, the intestinal origin in XVI, spermathecae in VII, and with segmental setal counts all greater than 40. *Pheretima bulusanensis***sp. nov.** has copulatory bursae openings 0.19–0.23 circumference apart and septa 8/9/10 present. *Pheretima sorsogonensis***sp. nov.** has copulatory bursae openings 0.14 circumference apart and septa 8/9/10 absent. *Pheretima blackbirdensis***sp. nov.** has copulatory bursae openings 0.17 circumference apart and septum 9/10 absent.

## ﻿Introduction

The Philippines archipelago is a biogeographically important region, home to some of the most diverse habitats in the world. Over half of the earthworm species known from the Philippines are found nowhere else in the world (Aspe et al. 2021). This estimate of endemism could be even higher for the largely neglected soil fauna. In particular, earthworm species exhibit high levels of endemism (Aspe and James 2014; Aspe et al. 2021; Decaëns et al. 2024), meaning that the overall endemism rate for Philippine earthworms is probably closer to 100% than to 50%.

Mount Bulusan is the southernmost volcano on Luzon Island in the Philippines. It is in the Sorsogon Province, approximately 250 km southeast of Manila on the Bicol Peninsula. Mount Bulusan is classified by volcanologists as a stratovolcano (composite volcano) and covers the northeast rim of the Irosin caldera that was formed approximately 40,000 years ago and has a peak elevation of 1,565 m above sea level.

Earthworm communities in Philippine forests are dominated by the genus *Pheretima*. Many Philippine species have been recently described, further expanding the diversity of this genus (Hong and James 2004, 2008a, 2008b, 2009, 2010, 2011a, 2011b, 2021; James 2004a, 2004b, 2006; James et al. 2004; Aspe and James 2014, 2015, 2016, 2017, 2018; Aspe et al. 2016, 2021). In this study, we describe three new earthworm species belonging to the *Pheretima sangirensis* group, characterized by a single pair of spermathecal pores in segment 7/8 (Sims and Easton 1972). A preliminary molecular phylogeny of Philippine *Pheretima* indicates that the included *sangirensis*-group species do form a clade, whereas all other provisional *Pheretima* species groups in the analysis were not monophyletic (Aspe and James 2018).

## ﻿Materials and methods

Earthworms were collected in Mount Bulusan on 3–4 May 2001 using digging, hand-sorting, inspecting leaf axils, and various other above-ground sites where humid organic matter accumulates. The collected specimens were euthanized in approximately 75% ethanol and fixed in 5–10% formaldehyde. Depending on the number of individuals per field-identifiable morphospecies, some were placed in 95% ethanol for molecular work. When the number of a morphospecies was limited (≤3), tissue samples were placed in ethanol before formaldehyde fixation. After 48 h, the formaldehyde-fixed specimens were transferred to 80% ethanol.

Morphological data were obtained through external examination and dorsal dissection from the head to mid-body under the microscope. Illustrations were prepared by freehand drawing and with a camera lucida. Holotype and paratypes were deposited at the National Museum of the Philippines Annelid collection (**NMA**).

## ﻿Taxonomic part

### ﻿Family Megascolecidae Rosa, 1891

**Genus *Pheretima* Kinberg, 1867**

#### ﻿ Pheretima bulusanensis

Taxon classificationAnimaliaCrassiclitellataMegascolecidae

Hong & James sp. nov.

476E6873-125E-544F-BBD1-641DF0FE64C9

https://zoobank.org/6BA85642-BCC6-4C6E-B51B-CD693F667988

Fig. 1

##### Type material.

***Holotype*.** Philippines • 1 clitellate; Sorsogon Province, Bulusan Volcano Natural Park, near a lake; 12°45.32'N, 124°04.34'E, 350–400 m a.s.l., 3 May 2001, S.W. James leg.; NMA 4802.

##### Additional material examined.

Philippines • 1 semi-clitellate; same data as for holotype • 1 clitellate; Sorsogon Province, Bulusan Volcano Natural Park, Aguinay crater edge; 12°46.6'N, 124°04.6'E; 792 m a.s.l.; 4 May 2001; S.W. James leg.

##### Type locality.

Philippines, Sorsogon Province, Bulusan Volcano Natural Park, near a lake (12°45.32'N, 124°04.34'E), 350–400 m.

##### Diagnosis.

*Pheretima* with one pair of spermathecal pores located deep in the intersegmental furrow between 7/8 at 10^th^ setal lines, equatorial unpigmented stripes, intestinal origin in XVI, spermathecae in VII, septa 8/9/10 present, all setal counts exceed 40, and seminal vesicle dorsal lobes are stalked and distinct from the main vesicle.

##### Description.

Dorsum violet-brown, ventrum pigmented I–IV, setal zones unpigmented widening ventrally, ventral XVIII–XX unpigmented; elsewhere light brown pigment adjacent to ventral intersegmental furrows. Dimensions 116–138 mm by 7 mm at segment X, 7.3 mm at XXX, 6.5 mm at clitellum, segments 97–104; body slightly oval in cross-section. Dorsal setae slightly larger and more widely spaced than ventral in pre-clitellar segments, setae numbering 43–47 at VII, 55–75 at XX; 10 between male pores, setal formula AA:AB:YZ:ZZ = 4:3:5:12 at XIII. Clitellum annular XIV–XVI; setae not visible externally. First dorsal pores 12/13. One pair of spermathecal pores deep in 7/8 at 10^th^ setal line, ventro-laterally placed, distance between spermathecal pores 5.3–6.0 mm, 0.18–0.27 circumference apart. Female pore single in XIV, transverse slit openings of copulatory bursae paired in XVIII at 9^th^ setal lines, ventrally placed, distance between openings 3.8–4.7 mm, 0.12–0.19 circumference apart. Genital markings lacking.

**Figure 1.**
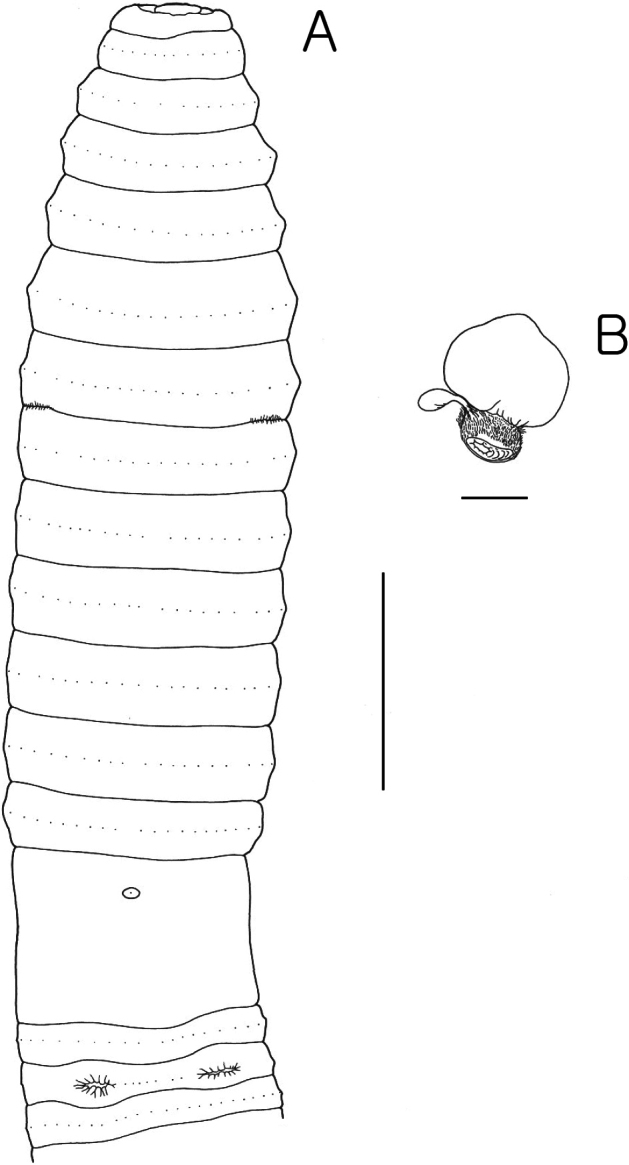
*Pheretima bulusanensis* sp. nov. A. Ventral view; B. Spermathecae. Scale bars: 7 mm (A); 1 mm (B).

Septa 5/6, 6/7 thick, 7/8 thin, 8/9/10 very thin, 10/11–13/14 thick. Gizzard in VIII–X, intestine begins in XVI, small pairs of lymph glands from XXVII along dorsal vessel; intestinal caeca simple originating in XXVII, and extending anteriorly about to XXIII, big finger-shaped sac; typhlosole low fold 1/6 lumen diameter. Hearts in X–XIII esophageal; X very small, IX lateral.

Ovaries and funnels in XIII, spermathecae one pair in VII, with nephridia on ducts; spermatheca with big globular or mushroom-shaped ampulla; stout barrel-shaped muscular, duct longer than ampulla, diverticulum with egg-shaped, iridescent chamber, stalk very short. Male sexual system holandric, testes and funnels in ventrally paired sacs in X–XI. Seminal vesicles two pairs in XI–XII, small stalked dorsal lobes attached in shallow clefts, prostates in XVII–XVIII, two or three lobes wrapped around copulatory bursae, muscular ducts hooked anteriorly to near center of copulatory bursae; copulatory bursae openings surrounded by toroid pad divided into four wedges; male pores on large, barrel-shaped penis from copulatory bursae roof.

##### Differential diagnosis.

*Pheretima bulusanensis* sp. nov. belongs to the *sangirensis* group of Sims and Easton (1972), which are characterized by a single pair of spermathecal pores in 7/8. The *P. sangirensis* group taxonomic history was reviewed by James (2004b). Based on a review of the recently published members of the group (James 2004b; Hong and James 2008a, 2011b, 2021; Aspe and James 2014, 2016, 2017) as well as several unpublished descriptions (Hong and James unpublished data), the present species can be distinguished from all other known species by the combination of equatorial unpigmented stripes, intestinal origin in XVI, spermathecae in VII, septa present in 8/9/10, and all setal counts being >40. Applying these filters, only this and the other two species described in this study are left for comparison. We observed that between *P. bulusanensis* sp. nov. and *P. sorsogonensis* sp. nov. the latter has fewer setae per segment throughout the body and between the male pores, closer genital pore spacing, lighter pigmentation that does not extend to the ventrum, a much smaller body size, and absence of septa 8/9/10. Even though the presence of septa was included in the “filter” list, this was currently disregarded due to it is difficult to observe under optimal dissection conditions. However, this character helps distinguish *P. blackbirdensis* sp. nov., which has septum 8/9. Other differences are that *P. blackbirdensis* sp. nov. has more setae in the preclitellar segments, somewhat closer genital pore spacing, seminal vesicle dorsal lobes that are apically (vs stalked in clefts) attached to the main vesicles, and prostatic ducts that enter the posterior face of the copulatory bursae rather than the center anterior. The two species differ slightly in size, with *P. blackbirdensis* sp. nov. being smaller.

##### Etymology.

The species is named for its type locality on Mount Bulusan.

#### ﻿ Pheretima sorsogonensis

Taxon classificationAnimaliaCrassiclitellataMegascolecidae

Hong & James sp. nov.

F8D18742-2D7D-58BF-9625-73DF635CE49D

https://zoobank.org/5F7C440E-E77B-42B2-B1E8-BA8FABF089D4

Fig. 2

##### Type material.

***Holotype*.** Philippines • 1 clitellate; Sorsogon Province, Bulusan Volcano Natural Park, near a lake; 12°45.32'N, 124°04.34'E; 350–400 m a.s.l.; 3 May 2001; S.W. James leg.; NMA 4803.

##### Additional material examined.

Philippines • 2 clitellates, 1 aclitellate; same data as for holotype.

##### Type locality.

Philippines, Sorsogon Province, Bulusan Volcano Natural Park, near a lake (12°45.32'N, 124°04.34'E), 350–400 m a.s.l.

##### Diagnosis.

*Pheretima* with one pair of spermathecal pores in 7/8 at 7^th^ setal line, equatorial unpigmented stripes, intestinal origin in XVI, spermathecae in VII, septa 8/9/10 absent, all setal counts greater than 40, 4–6 setae between male pores, and seminal vesicle dorsal lobes apically attached, elongate with wide heads.

##### Description.

Medium light-brown dorsal pigment, with thin pale zones at setal rings. Dimensions 56–68 mm by 3.0–3.5 at segment X, 3.3–3.4 mm at XXX, 3.0–3.5 mm at clitellum, segments 97–108; body circular in cross-section. Setae numbering 39–42 at VII, 45–50 at XX; 4–6 between male pores, slightly more crowded ventrally, setal formula AA:AB:YZ:ZZ = 2.5:2:3:4 at XIII. Clitellum annular XIV–XVI, setae not visible externally. First dorsal pores 12/13. One pair large spermathecal pore in 7/8 at 7^th^ setal line, overhanging lip from VII slightly lateral to pore, distance between spermathecal pores around 1.6–1.8 mm, 0.11–0.15 circumference apart. Female pore single in XIV, 0.3–0.4 mm openings of copulatory bursae paired in XVIII at 5^th^ setal line, ventral, distance between 1.4–1.5 mm, 0.11–0.13 circumference apart. Genital markings lacking.

**Figure 2.**
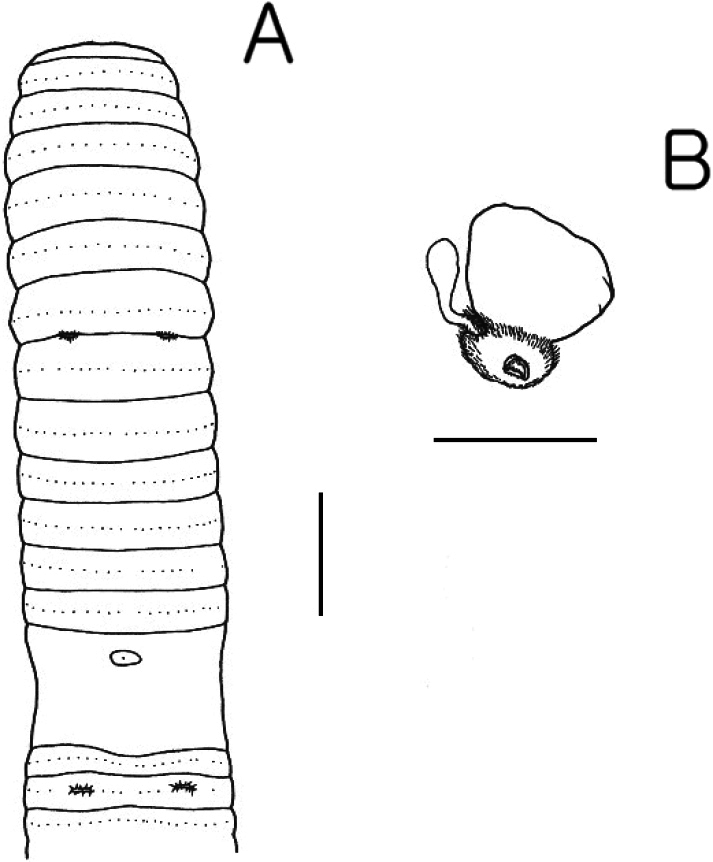
*Pheretima sorsogonensis* sp. nov. A. Ventral view; B. Spermathecae. Scale bars: 5 mm (A); 1 mm (B).

Septa 5/6, 6/7, 7/8 muscular but not thick, 8/9, 9/10 absent, 10/11–12/13 moderately thick. Gizzard in VIII–X. Esophagus with chevron lamellae XI–XIII; intestine begins in XVI; small, paired lymph glands from XXVII along dorsal vessel; intestinal caeca simple originating in XXVII, and extending anteriorly about to XXIV, tapered sac with some pockets on ventral margins; typhlosole very small found from XXVII, 30 intestinal blood vessels. Hearts in X–XIII esophageal.

Ovaries and funnels in XIII, spermathecae one pair in VII, with nephridia on ducts; spermatheca with thick lenticular ampulla, weakly muscular duct shorter than ampulla; diverticulum chamber small, egg-shaped, iridescent pearl-colored, stalk slender, one or two kinks close to duct. Male sexual system holandric, testes and funnels in ventrally paired sacs in X–XI. Seminal vesicles two pairs in XI–XII, dorsal lobes apical, elongate with wide heads, prostates in XVII–XX, glands 2–3 lobes wrapped around copulatory bursae, muscular ducts form U-shape concave anteriorly on posterior of copulatory bursae lacking stalked glands; duct enters center of copulatory bursae; copulatory bursae openings surrounded toroid pad divided into four wedges, penis triangular block with apical pore.

##### Differential diagnosis.

*Pheretima sorsogonensis* sp. nov. has been distinguished from *P. bulusanensis* sp. nov. and all other known members of the *sangirensis* group. The final comparison is with *P. blackbirdensis* sp. nov., from which it differs in having a smaller body size yet greater number of setae, fewer setae between the male pores, and far fewer intestinal blood vessels. In terms of spermathecae, the *P. sorsogonensis* sp. nov. has weak muscular ducts much shorter than the ampulla, whereas *P. blackbirdensis* sp nov. has muscular ducts that are nearly as long as the ampulla.

##### Etymology.

The species is named for its type locality in Sorsogon Province.

#### ﻿ Pheretima blackbirdensis

Taxon classificationAnimaliaCrassiclitellataMegascolecidae

Hong & James sp. nov.

70E44481-BE07-5208-B7B0-10B426E058B8

https://zoobank.org/4CAF8238-0334-4587-9294-B1CCBE422DCD

Fig. 3

##### Type material.

***Holotype*.** Philippines • 1 clitellate; Sorsogon Province, Bulusan Volcano Natural Park, at large dry crater bed called Aguinay; 12°46.60'N, 124°04.60'E; 792 m a.s.l.; 4 May 2001; S.W. James leg.; NMA 4804.

##### Additional material examined.

Philippines • 2 clitellates, same data as for holotype.

##### Type locality.

Philippines, Sorsogon Province, Bulusan Volcano Natural Park, at large dry crater bed called Aguinay (12°46.60'N, 124°04.60'E), 792 m a.s.l.

##### Diagnosis.

One pair spermathecal pores deep in 7/8 at 10^th^ setal line, equatorial unpigmented stripes, intestinal origin in XVI, spermathecae in VII, septum 8/9 present, all setal counts greater than 40, long muscular spermathecal ducts, seminal vesicle dorsal lobes triangular not differentiated from main vesicles.

##### Description.

Dorsal pigment medium purple-brown, absent from setal rings; unpigmented stripes interrupted at dorsal setal gaps. Dimensions 85 mm by 8 at segment X, 7 mm at XXX, 7.5 mm at clitellum, segments 62; body oval in cross-section. Setae numbering 52 at VII, 60 at XX, 14 between male pores, spacing greater in dorsum, setal formula AA:AB:YZ:ZZ = 3:2.5:12:3.5 at XIII. Clitellum annular XIV–XVI, setae not visible externally. First dorsal pores 11/12. One pair of spermathecal pores deep in 7/8 at 10^th^ setal lines, sub-lateral, distance between spermathecal pores 5 mm, 0.20 circumference apart. Female pore single in XIV, openings of copulatory bursa paired in XVIII centered between 10^th^–11^th^ setal lines, 4 mm between openings, 0.17 circumference apart. Genital markings lacking.

**Figure 3.**
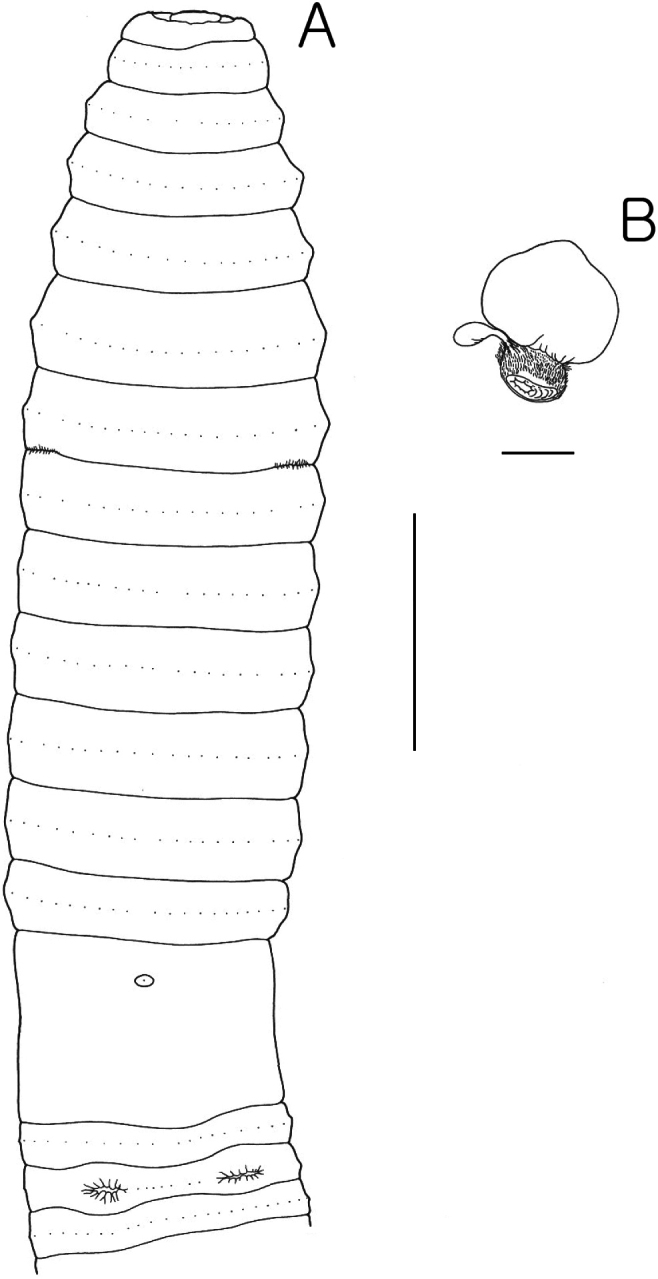
*Pheretima blackbirdensis* sp. nov. A. Ventral view; B. Spermathecae; Scale bars: 10 mm (A); 1 mm (B).

Septa 5/6–7/8 thinly muscular, 8/9 membranous, 9/10 absent, 10/11–13/14 thinly muscular. Gizzard in VIII, intestine begins in XVI, paired lymph glands from XXVIII along dorsal vessel; intestinal caeca simple originating in XVII, and extending anteriorly about to XXIII, finger-shaped sac; typhlosole small from XXVII. Hearts in X–XIII esophageal, 42–44 intestinal blood vessels.

Ovaries and funnels in XIII, spermathecae one pair in VII, with nephridia on ducts, wrinkled spherical ampulla; duct very muscular, broad based bulbous cone almost as long as ampulla, diverticulum chamber broad asymmetrical oval, stalk shorter than ampulla. Several spherical spermatophores with long tails in each ampulla. Male sexual system holandric, testes and funnels in ventrally paired sacs in X–XI. Seminal vesicles two pairs in XI–XII, medium-sized with triangular dorsal lobes not distinctly marked from main vesicle, prostates in XVII–XVIII, with muscular ducts curving around posterior of copulatory bursae, entering posterior face of copulatory bursae without stalked glands; copulatory bursae openings surrounded by torus-shaped pad incompletely divided into four or five unequal wedges; penis a short cylinder with central conical protrusion bearing apical male pore.

##### Differential diagnosis.

*Pheretima blackbirdensis* sp. nov. differs from the other two new species described above, including their spermathecal morphology. Comparing the spermathecal ducts, *P. sorsogonensis* sp. nov. has weak muscular ducts much shorter than the ampulla, whereas *P. blackbirdensis* sp. nov. has ducts that are very muscular and nearly as long as the ampulla.

##### Remarks.

This species is intermediate in size between the other two described here but has far fewer segments (62 vs 100 or so). However, it cannot be confirmed whether the specimens had lost some segments to predators.

##### Etymology.

The species is named for Lake Blackbird, which is close to the type locality.

## Supplementary Material

XML Treatment for Pheretima bulusanensis

XML Treatment for Pheretima sorsogonensis

XML Treatment for Pheretima blackbirdensis

## ﻿Additional information

### Use of AI

No use of AI was reported.

### Author ORCIDs

Yong Hong https://orcid.org/0000-0002-8093-9717

Samuel W. James https://orcid.org/0000-0001-6758-5726

### Data availability

All of the data that support the findings of this study are available in the main text.

## References

[B1] Aspe NM James SW (2014) New species of *Pheretima* (Oligochaeta: Megascolecidae) from the Mt. Malindang Range, Mindanao Island, Philippines. Zootaxa 3881(5): 401–439. 10.11646/zootaxa.3881.5.1 25543645

[B2] Aspe NM James SW (2015) New *Polypheretima* and *Pithemera* (Oligochaeta: Megascolecidae) species from the Mt. Malindang Range, Mindanao Island, Philippines. Journal of Natural History 49(37–38): 2233–2255. 10.1080/00222933.2015.1021875

[B3] Aspe NM James SW (2016) New species of *Pheretima*, *Amynthas*, *Polypheretima*, and *Pithemera* (Clitellata: Megascolecidae) from Mindanao and associated islands, Philippines. Zoological Studies (Taipei, Taiwan) 55: 8–49. 10.1080/00222933.2015.1021875 PMC6511825 31966153

[B4] Aspe NM James SW (2017) Pheretimoid earthworms (Clitellata: Megascolecidae) from Mt. Apo, Mindanao Island, Philippines with description of eight new species. The Raffles Bulletin of Zoology 65: 357–372.

[B5] Aspe NM James SW (2018) Molecular phylogeny and biogeographic distribution of pheretimoid earthworms (Clitellata: Megascolecidae) of the Philippine archipelago. European Journal of Soil Biology 85: 89–97. 10.1016/j.ejsobi.2018.02.001

[B6] Aspe NM Kajihara K James SW (2016) A molecular phylogenetic study of pheretimoid species (Megascolecidae) in Mindanao and associated islands, Philippines. European Journal of Soil Biology 73: 119–125. 10.1016/j.ejsobi.2016.02.006

[B7] Aspe NM Manasan RE Manlavi AB Patiluna MLE Sebido MAB Obusan MCM Simbahan JF James SW (2021) The earthworm fauna of Palawan, Philippines with description of nineteen new pheretimoid species (Clitellata: Megascolecidae). Journal of Natural History 55(11–12): 733–797. 10.1080/00222933.2021.1923849

[B8] Decaëns T Bartz MLC Feijoo-Martínez A Goulpeau A Lapied E Marchán DF Maggia M-E Papuga G James SW (2024) Earthworms (Oligochaeta, Clitellata) of the Mitaraka range (French Guiana): Commented checklist with description of one genus and eighteen species new to science. Zoosystema 46(9): 196–244. 10.5252/zoosystema2024v46a9

[B9] Hong Y James SW (2004) New species of *Amynthas* Kinberg, 1867 from the Philippines (Oligochaeta: Megascolecidae). Revue Suisse de Zoologie 111: 729–741. 10.5962/bhl.part.80266

[B10] Hong Y James SW (2008a) Three new earthworms of the genus *Pheretima* (Oligochaeta: Megascolecidae) from Mt. Makiling, Luzon Island, Philippines. Zootaxa 1695(1): 45–52. 10.11646/zootaxa.1695.1.2

[B11] Hong Y James SW (2008b) Two new earthworms of the genus *Pheretima* (Oligochaeta: Megascolecidae) from Mt. Isarog, Luzon Island, Philippines. Journal of Natural History 42(23–24): 1565–1571. 10.1080/00222930802000398

[B12] Hong Y James SW (2009) New earthworms of the *Pheretima urceolata* species group (Oligochaeta: Megascolecidae) from southern Luzon, Philippines. Zootaxa 2059(1): 33–45. 10.11646/zootaxa.2059.1.3

[B13] Hong Y James SW (2010) Six new earthworms of the genus *Pheretima* (Oligochaeta: Megascolecidae) from Balbalan-Balbalasang, Kalinga Province, Philippines. Zoological Studies (Taipei, Taiwan) 9: 523–533.

[B14] Hong Y James SW (2011a) New earthworms of *Pheretima*, *Pithemera*, and *Polypheretima* (Clitellata: Megascolecidae) from Kalbaryo, Luzon Island, Philippines. The Raffles Bulletin of Zoology 59: 19–28.

[B15] Hong Y James SW (2011b) New earthworm species of the genus *Pheretima* (Clitellata: Megascolecidae) from Mountain Province, Philippines. Journal of Natural History 45(29–30): 1769–1788. 10.1080/00222933.2011.560726

[B16] Hong Y James SW (2021) Two new earthworms of the *Pheretima sangirensis* species group (Clitellata: Megascolecidae) from Mountain Province, Philippines. Zootaxa 4995(2): 357–366. 10.11646/zootaxa.4995.2.8 34810566

[B17] James SW (2004a) New genera and new species of earthworms (Clitellata: Megascolecidae) from southern Luzon, Philippines. Systematics and Biodiversity 2(3): 271–279. 10.1017/S1477200004001446

[B18] James SW (2004b) New species of *Amynthas*, *Pheretima*, and *Pleionogaster* (Oligochaeta: Megascolecidae) of the Mt. Kitanglad Range, Mindanao Island, Philippines. Raffles Bulletin of Zoology, Singapore 52: 289–313.

[B19] James SW (2006) The earthworm genus *Pleionogaster* (Clitellata: Megascolecidae) in southern Luzon, Philippines. Organisms Diversity & Evolution 6(3): 167–170. [Electronic Supplement 8: 1–20.] 10.1016/j.ode.2005.08.003

[B20] James SW Hong Y Kim TH (2004) New earthworms of *Pheretima* and *Pithemera* (Oligochaeta: Megascolecidae) from Mt. Arayat, Luzon Island, Philippines. Revue Suisse de Zoologie 111: 3–10. 10.5962/bhl.part.80221

[B21] Sims RW Easton EG (1972) A numerical revision of the earthworm genus *Pheretima* auct. (Megascolecidae: Oligochaeta) with the recognition of new genera and an appendix on the earthworms collected by the Royal Society North Borneo Expedition. Biological Journal of the Linnean Society. Linnean Society of London 4(3): 169–268. 10.1111/j.1095-8312.1972.tb00694.x

